# Atorvastatin-Induced Necrotizing Myopathy and its Response to Combination Therapy

**DOI:** 10.7759/cureus.12957

**Published:** 2021-01-28

**Authors:** Sri Harsha Boppana, Hasan A Syed, Daniel Antwi-Amoabeng, Prahlad Reddy, Nageshwara Gullapalli

**Affiliations:** 1 Internal Medicine, University of Nevada, Reno, Reno, USA; 2 Internal Medicine, University of Nevada Reno School of Medicine, Reno, USA; 3 Rheumatology, Arthritis Consultants, Reno, USA

**Keywords:** atorvastatin, myopathy

## Abstract

Atorvastatin is the most commonly used statin medication to decrease cholesterol levels and prevent atherosclerosis. Myopathy is a reported side effect of atorvastatin which can happen even after more than six months after starting the medication. The side effect on the muscle tissue can range from simple reversible myalgia to respiratory muscle compromise. Here we present a 46-year-old male who presented with myopathy after taking atorvastatin for two years. Biopsy proved immune-mediated necrotizing myopathy which responded to a combination of Rituximab and intravenous immunoglobulin therapy.

## Introduction

Statins are the most used medications for lowering cholesterol levels which act by inhibiting the HMG Co-A reductase enzyme. Although rare, myocyte damage is reported in 1.5%-5% population as a side effect of this medication through immune destruction or non-immune destruction of myocyte [1]. Muscle damage can present as simple myalgia, dysphagia, or life-threatening muscle weakness with resultant respiratory mechanics dysfunction [2]. Statin use has been associated with autoimmune antibody development against muscle fiber enzymes causing myopathy [3]. It is marked by elevations in creatine kinase levels and biopsy often reveal necrosis of the muscle [4]. The Statin-Associated Muscle Symptom Clinical Index (SAMS-CI) was a tool proposed to attribute statin as a cause of myopathy [5]. Here, we present a case of atorvastatin-induced necrotizing myopathy.

## Case presentation

A 46-year-old non-Hispanic male with a history of hypertension, type 2 diabetes, and dyslipidemia was admitted to the hospital with complaints of progressive proximal muscle weakness for three months. He has been taking atorvastatin for two years before the presentation. Initially, he had difficulty raising his arms above the shoulder for two to three months prior to presentation, but this progressed to difficulty climbing stairs. Vitals signs were within normal limits. Physical exam was significant for erythematous rash seen on bilateral cheeks but sparing the eyes. Weakness was seen predominantly in the proximal musculature involving deltoids, bilateral quadriceps, and hamstrings. However, knee extension/flexion and foot dorsiflexion appear spared. Labs showed creatinine phosphokinase levels elevated to more than 22,000 units/L and aspartate transaminase /alanine transaminase were 420/410 units/L with normal renal function.

**Figure 1:**
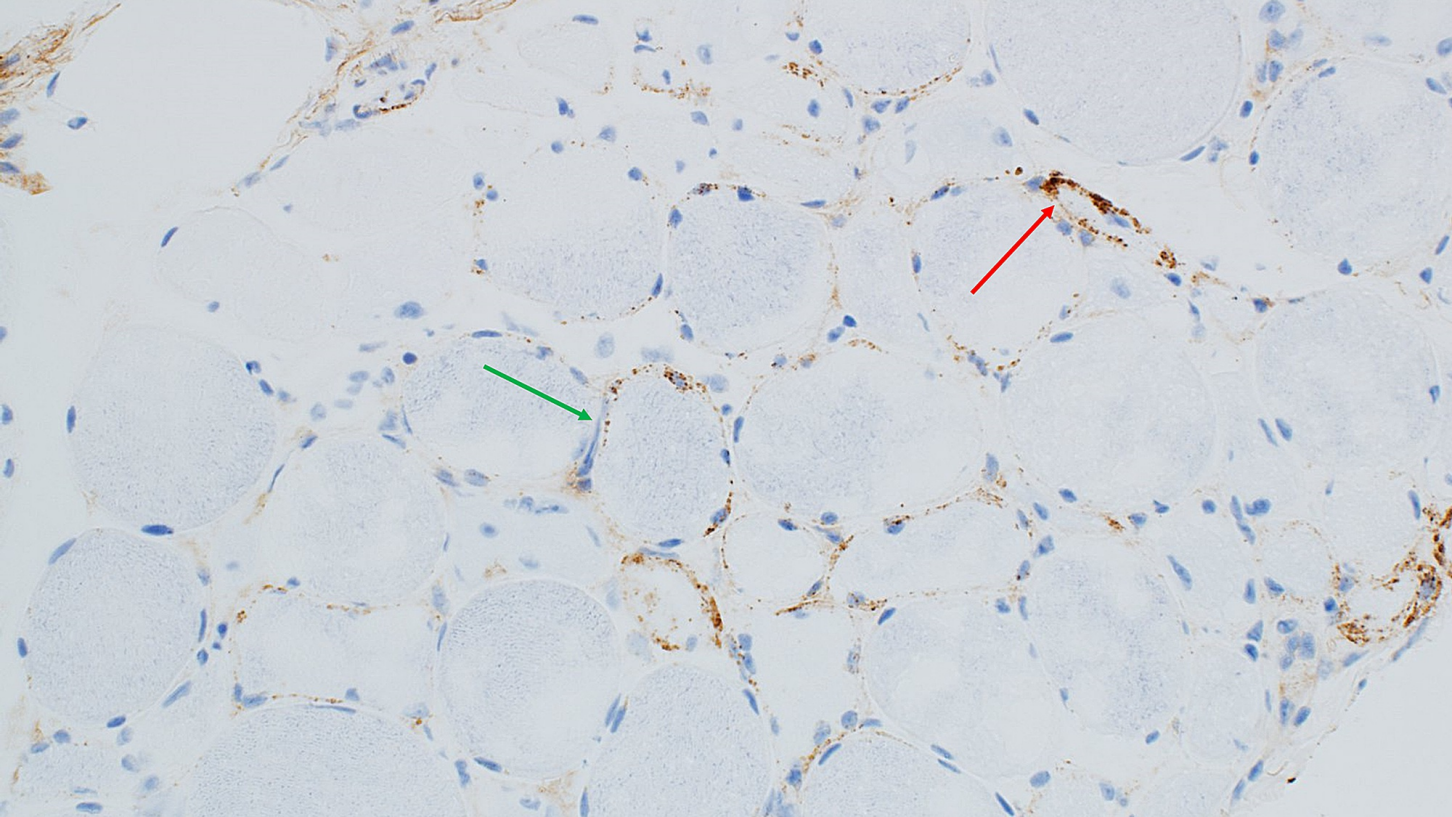
C5b9 immunohistochemistry; 200X- C5b9 staining endomyseal capillaries (red arrow) and granular sarcolemmal staining (green arrow).

**Figure 2:**
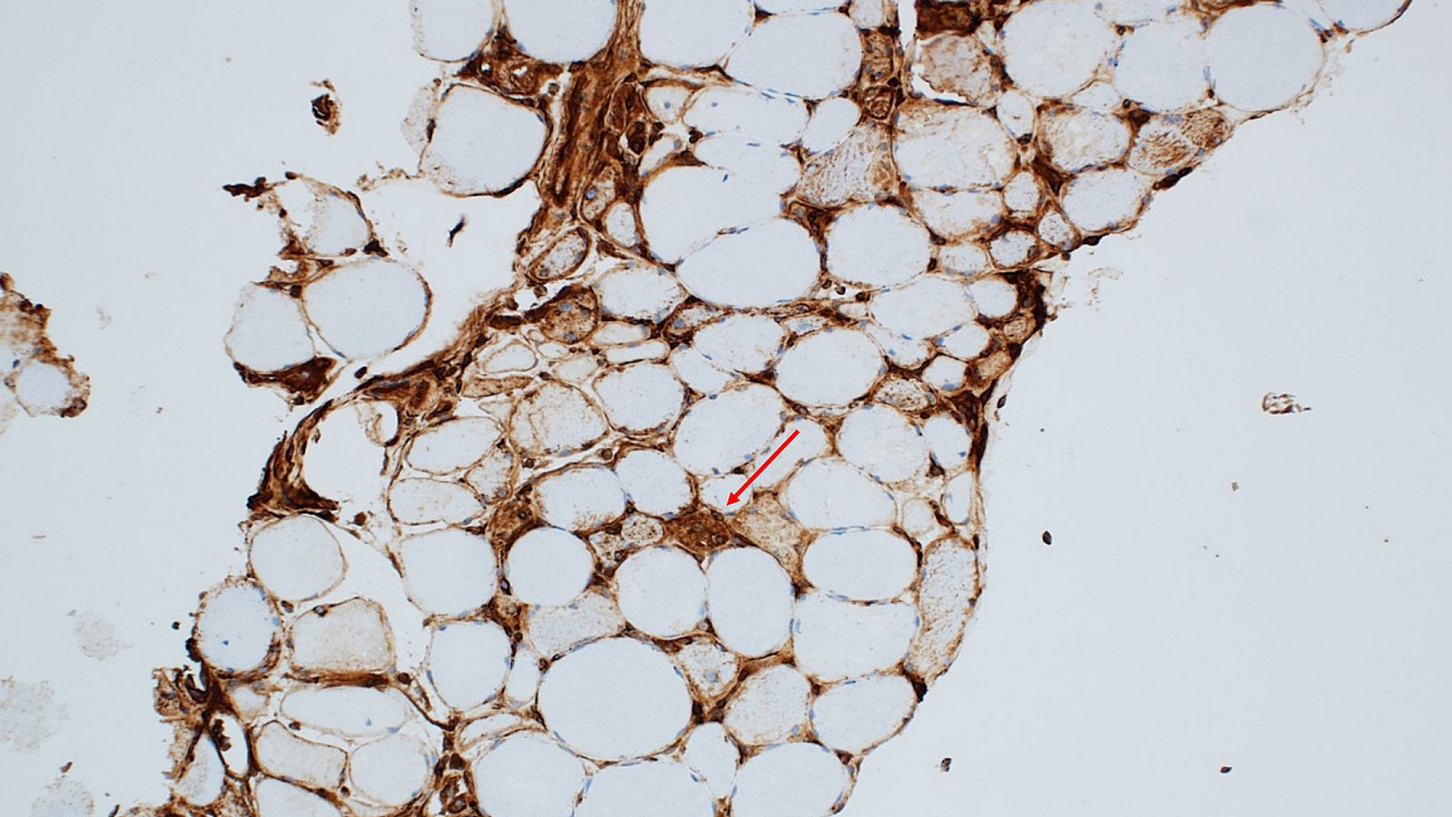
MHC1 immunohistochemistry; 200X- MHC1 staining regenerating nerve fiber (red arrow).

Neurology was consulted who recommended electromyography (EMG) and muscle biopsy. EMG demonstrated widespread myopathy. Left gluteus maximus muscle biopsy showed 3-hydroxy-3 methyl-glutaryl-coenzyme A reductase (HMGCR) related to immune-mediated necrotizing myopathy (Figures 1, 2). He also had an elevation of 3-hydroxy-3 methyl-glutaryl-coenzyme A reductase (HMGCR) IgG antibody to more than 200 units (normal: 0-19 units). Statin medication was discontinued on admission and patient was started on oral prednisone 80 mg for immune-mediated necrotizing myopathy. He had mild improvement in his muscle strength along with a slight decrease in his CPK levels to the range of 10,000-13,000 units/L after two weeks of use. He was also started on Bactrim for pneumocystis pneumonia prophylaxis considering long-term high dose steroid administration.

He was discharged three weeks after his initial admission with oral prednisone 80 mg daily and outpatient rheumatology follow up. During his outpatient rheumatology visit, prednisone was decreased to 40 mg daily and he was started on methotrexate. He was admitted to the hospital after two weeks of treatment with methotrexate complaining of worsening muscle weakness along with declining motor skills. His creatinine phosphokinase (CPK) was 10912 U/L, aspartate aminotransferase/alanine aminotransferase was 486/532 units/L. One gram of intravenous methylprednisolone was administered for three days and followed by intravenous immunoglobulin for two days as per rheumatology recommendations. He was discharged to inpatient rehabilitation after six days of a hospital stay along with a recommendation to continue prednisone 40 mg and discontinuation of methotrexate.

He was re-admitted to the hospital from the rehabilitation facility after three days with complaints of shortness of breath, difficulty coughing, and difficulty clearing his oral secretions. On arrival to the emergency room, he was found to be hypoxic, saturating at 89% on 15 liters/minute oxygen support via a nonrebreather mask. He was admitted to the ICU considering his increasing oxygen requirements. He was transitioned to high-flow nasal cannula 50 litres, 100% oxygen with improvement in saturations to a low 90% range. His respiratory status further deteriorated secondary to neuromuscular respiratory failure. He was intubated and mechanically ventilated. Later, was started on 500 mg intravenous methylprednisolone for three days with subsequent tapering. Rheumatology was reconsulted who recommended starting him on the rituximab regimen for four weeks showing moderate improvement in muscle weakness. He was eventually weaned off to percutaneous tracheostomy and discharged to a long-term acute care facility. He was transferred to a rehabilitation facility. He continued to receive intravenous immunoglobulin 75 g once a week. Thereafter he has shown improvement in his muscle weakness and downtrend of creatinine phosphokinase to 1,193 units/L.

## Discussion

Statin use has been strongly associated with HMG CR Ab positivity in idiopathic inflammatory myopathy patients [6]. In patients with idiopathic inflammatory myopathy, a strong correlation between necrotizing autoimmune myopathy and detection of 3-hydroxy-3-methylglutaryl-coenzyme A reductase autoantibodies in patients exposed to statin. Antibody titers were proportional to the degree of muscle destruction [7].

Environmental along with genetic risk factors (human leukocyte antigen D-related B 111: 01) is associated with statin-induced immune-mediated necrotizing myopathy (IMNM) [8]. IMNM was more common in atorvastatin exposure compared to other statins. Other associated risk factors are female gender and old age with more positive anti-HMG CR rate [9]. IMNM is characterized by anti-HMG CR antibody or anti-SRP positivity in more severe form, than Ab negative with early treatment resulting in better outcomes [10,11]. Anti-HMG CR was strongly predictive in the diagnosis of statin-induced IMNM [12,13]. However, antibodies are not specific to IMNM in statin exposed versus unexposed patients [14]. The downtrend of these antibodies and creatinine kinase levels has indicated improvement in muscle weakness [15]. After stopping the statin; during muscle regeneration, high levels of expression HMG CR can still contribute to immune response [16]. Statin-induced anti-HMG CR is mainly confirmed using enzyme-linked immunosorbent assays (ELISAs)/immunoblot [17]. There are few case reports with successful treatment of IMNM with steroids, methotrexate and intravenous immunoglobulin [16,18]. In small Australian case series, early steroid withdrawal resulted in relapses of muscle weakness. These patients required other modalities of immunosuppression including IVIG, methotrexate, cyclophosphamide and rituximab [19]. Rituximab treatment in nine patients has shown better clinical improvement in terms of muscle strength in resistant anti-HMG CR-mediated IMNM [20].

## Conclusions

The use of statin therapy has been very common in medical practice to prevent atherosclerotic disease. Our patient presented with myopathy from statin use after almost two years of use of this medication with SAMS-CI score of six. Muscle weakness can lead to life-threatening respiratory failure. We recommend re-conciliation of the medication list for statin therapy, even though it is rarely observed as a late side effect. In this patient, we have tried all the modalities of treatment including pulse dose steroids and methotrexate. Treatment with a combination of rituximab and intravenous immunoglobulin, the patient had improved muscle strength along with the downtrend of creatinine phosphokinase.
